# Automated ancillary cancer history classification for mesothelioma patients from free-text clinical reports

**DOI:** 10.4103/2153-3539.71065

**Published:** 2010-10-11

**Authors:** Richard A. Wilson, Wendy W. Chapman, Shawn J. DeFries, Michael J. Becich, Brian E. Chapman

**Affiliations:** Department of Biomedical Informatics, University of Pittsburgh, 200 Meyran Avenue, Pittsburgh, PA; USA; 1 Department of Biomedical Informatics, Keller Army Community Hospital, 900 Washington Road, West Point, NY, USA

**Keywords:** Information extraction, natural language processing, cancer history classifcation

## Abstract

**Background:**

Clinical records are often unstructured, free-text documents that create information extraction challenges and costs. Healthcare delivery and research organizations, such as the National Mesothelioma Virtual Bank, require the aggregation of both structured and unstructured data types. Natural language processing offers techniques for automatically extracting information from unstructured, free-text documents.

**Methods:**

Five hundred and eight history and physical reports from mesothelioma patients were split into development (208) and test sets (300). A reference standard was developed and each report was annotated by experts with regard to the patient’s personal history of ancillary cancer and family history of any cancer. The Hx application was developed to process reports, extract relevant features, perform reference resolution and classify them with regard to cancer history. Two methods, Dynamic-Window and ConText, for extracting information were evaluated. Hx’s classification responses using each of the two methods were measured against the reference standard. The average Cohen’s weighted kappa served as the human benchmark in evaluating the system.

**Results:**

Hx had a high overall accuracy, with each method, scoring 96.2%. F-measures using the Dynamic-Window and ConText methods were 91.8% and 91.6%, which were comparable to the human benchmark of 92.8%. For the personal history classification, Dynamic-Window scored highest with 89.2% and for the family history classification, ConText scored highest with 97.6%, in which both methods were comparable to the human benchmark of 88.3% and 97.2%, respectively.

**Conclusion:**

We evaluated an automated application’s performance in classifying a mesothelioma patient’s personal and family history of cancer from clinical reports. To do so, the Hx application must process reports, identify cancer concepts, distinguish the known mesothelioma from ancillary cancers, recognize negation, perform reference resolution and determine the experiencer. Results indicated that both information extraction methods tested were dependant on the domain-specific lexicon and negation extraction. We showed that the more general method, ConText, performed as well as our task-specific method. Although Dynamic- Window could be modified to retrieve other concepts, ConText is more robust and performs better on inconclusive concepts. Hx could greatly improve and expedite the process of extracting data from free-text, clinical records for a variety of research or healthcare delivery organizations.

## INTRODUCTION

The current healthcare and research environment requires the aggregation of various types of medical data. These data types include structured data elements like laboratory values, patient age and International Classification of Disease (ICD) codes, and are easily queried from other systems. Medical data types also include unstructured data such as clinical reports, which describe clinical encounters in the narrative form as in discharge summary or pathology report. These free-text, clinical reports contain a plethora of information, but create data extraction challenges. As a result, these reports must be manually abstracted at a great cost of time and resources. Alternatively, natural language processing (NLP) offers techniques for automatically extracting information from free-text documents.[1]

Healthcare delivery, registry and clinical research organizations require the aggregation of both structured and unstructured data types. For example, the National Mesothelioma Virtual Bank (NMVB) is a virtual tissue and biospecimen repository and registry designed to facilitate clinical and translational research for mesothelioma. NMVB centralizes a large collection of mesothelioma-related specimens into a single “clinical annotation engine.”[2] Its goal is to aggregate research resources in order to accelerate pathophysiologic and clinical treatment discoveries for mesothelioma. To date, the NMVB contains 837 annotated cases and 1,014 biospecimens of paraffin-embedded tissue, fresh frozen tissue and blood and DNA samples.[2]

Mesothelioma is a cancer of the mesothelium, the membrane in the lining of the chest or abdomen. This disease has a fairly low incidence rate of about 3,000 cases per year in the US, and it affects five-times more men than women. Although the incidence is low, mesothelioma is highly fatal and has a 5-year survival rate of only 7.9%.[3] The US epidemiologic projections predict that current rates for males have peaked and will begin a decline over the next 40 years.[4] However, Europe appears be in the middle of an epidemic, with mortality rates steadily rising and expected to more than double by 2015–19 to almost 7,000 deaths per year.[5] Presently, treatment for this disease generally involves the “trimodality therapy,” consisting of surgery (e.g., pneumonectomy), radiotherapy and chemotherapy.[6] Many believe that this disease is untreatable and warrants only palliative support and care.[7] Mesothelioma is directly linked to asbestos exposure in approximately 95% of the cases and, therefore, is highly litigated.[7] Low-incidence rates and litigation issues have limited the number of cases available for banking. Research in this field, and ultimately patients, would benefit greatly from a significant increase in the number of abstracted cases available for study, including the incorporation of more clinical information from patient records. Such radical expansion of cases for any databank or registry would require implementation of automated data collection and information extraction tasks to be completed.

Currently, tissue banks and registries often rely on data abstracted manually from free-text medical records. Abstracting data from these reports is costly in terms of time, personnel and money. Most medical researchers know the far-too familiar scene of staff combing through stacks of medical records gathering data. Data collection is timely and tedious: collection instruments and guidelines must be developed; training for staff must be produced and implemented to limit bias in the data set; and staff must be dedicated to the task and/or diverted from other opportunities. Selecting the right type of staff members (e.g., physicians, coders, nurses) to perform the mission can also affect the accuracy and cost of the data set.[8] For example, each case entered into the NMVB is abstracted for 140 common data elements, including demographic, epidemiologic, pathologic, genotype and follow-up data. The patient’s personal and family history of cancer is one important data element to be abstracted. Specifically, researchers are interested in the patient’s history of ancillary cancers (not mesothelioma) and the family history of any form of malignancy. Although this information is commonly contained in free-text clinical reports, NLP may provide automated methods to retrieve this information.

Computer applications have long been applied to processing structured data elements such as account balances or patient temperature values. However, much of a patient’s clinical record is an unstructured, free-text form. Clinical records such as history and physicals (HandPs), progress notes and discharge summaries narrate the patient’s clinical course in our natural language (e.g., “Patient presented to the ED today complaining of severe chest discomfort, but without an elevated heart rate.”). NLP provides a means to convert our natural language in the form of unstructured text into structured data elements (e.g., heart rate = normal). To structure the data, NLP applications perform a number of core tasks. Tokenization separates strings of electronic prose into individual words, or tokens. Part of speech tagging works to reduce ambiguity, such as the differences in the word *patient* as a noun (*the patient is sick*) or as an adjective (*she is a patient woman*). The syntax of specific text is analyzed to understand the grammar rules that define how words combine to form phrases/sentences. Context is also analyzed in order to evaluate phrases/sentences in connection with the surrounding text. Semantics works to map tokens to concepts found in a supporting lexicon or ontology (e.g., UMLS). NLP can then be applied to information extraction, classification, search or translation tasks.[9]

NLP can be a particularly difficult task due to the intricacy of language and the ambiguous nature of individual words or phrases. Language is filled with synonyms, homographs, abbreviations, jargon, vague references, misspellings, ambiguity and domain-specific terms. Application of NLP in the medical domain generates its own set of unique challenges. Medical NLP systems must be highly sensitive and specific, interpret knowledge commonly assumed in compact medical documents, overcome lack of access to annotated sets of clinical records due to privacy safeguards and manage a large set of specialized clinical domains, each with its own particular terminology.[9]

Reference resolution is a common problem in all NLP domains. Natural language contains many pronouns, noun phrases, demonstratives (e.g., this dog or that cat) and names that refer to some previously established reference. Consider the following passage: *The patient complains of severe chest pain. His EKG was unremarkable*. In order to comprehend this full passage, it is imperative to resolve that *“His”* is referring to *“patient”* and not *“chest,” “pain”* or some other entity from a previous sentence. The human brain resolves this reference routinely, but it is not uncommon for one to be confused as to which subject the author is referring. Computer applications must be taught to resolve reference problems as well.[1]

NLP applications have been tailored to extract data and classify documents with ever-improving accuracy. These applications have used a variety of tools and techniques to accomplish their task. Frames are a tool used quite extensively to capture semantic concepts and its features. The concept features, called slots, hold values extracted from the text (e.g., Patient: Temperature = 98.6).[1] Hot-spotting is a technique by which identified locations of interest in the text serve as a base for additional search and extraction of feature values from the surrounding text.[10] Domain-specific lexical knowledge sources have proved necessary when dealing with the language of clinical documents.[11] Finally, leveraging either generated or existing clinical record organization such as section headers can improve information extraction performance.[12] NLP has provided techniques for information retrieval from text, but the task itself remains challenging.

Our study aims to test the following hypothesis: *an automated NLP application can classify personal and family history from free-text reports as well as a human*. Specifically, the application would be judged in its ability to classify a report with regard to two primary questions: Q1 – does the patient have a personal history of cancer, ancillary to the known mesothelioma?; and, Q2 – does the patient have a family history of any cancer? What makes this task particularly challenging is that the reports come from a clinical subpopulation of mesothelioma patients. The application must not only identify relevant concepts in the text but must also (1) attribute the cancer to the patient or a family member and (2) resolve whether the concept being described is the known cancer (e.g., mesothelioma) or an ancillary cancer.

## METHODS

To test our hypothesis, we compared the results of an NLP application against a human-annotated reference standard on a test set of HandP reports. After receiving Institutional Review Board approval, we randomly selected 120 patients from the University of Pittsburgh Medical Center (UPMC), Medical Archival System (MARS), with a positive surgical pathology report of mesothelioma and a hospital admission occurring after the patient’s mesothelioma diagnosis.[13] The reports from this subpopulation of mesothelioma patients were then deidentified using the locally developed software, *De-ID*.[14] Our task is at the report level. Therefore, multiple reports from an individual patient were included, but were limited to no more than 10 reports per any one patient. The result was a data set of 508 free-text HandP reports, which were then broken into development (training) and testing sets of 208 and 300 reports, respectively.

The reference standard was created by human annotators and served as the correct classification when evaluating the application responses for each report. In order to minimize subjectivity and individual tendencies, the majority response of three annotators was used as the reference standard. Our annotation team included a physician, a health information professional and a biomedical informatician. Annotation guidelines were developed and training was conducted prior to beginning annotation. Annotators were asked to read each HandP report and answere two primary questions: Q1 – does the patient have a history of cancer, ancillary to the known mesothelioma; and, Q2 – does the patient have a family history of any cancer. Although two data elements were evaluated in this study, seven primary data elements were annotated for future work. The other elements included the types of cancer identified, first-degree relatives with cancer and/or mesothelioma and non-first-degree relatives with mesothelioma. After annotation of the 300 HandPs was completed, agreement was measured.

**Figure 1:**
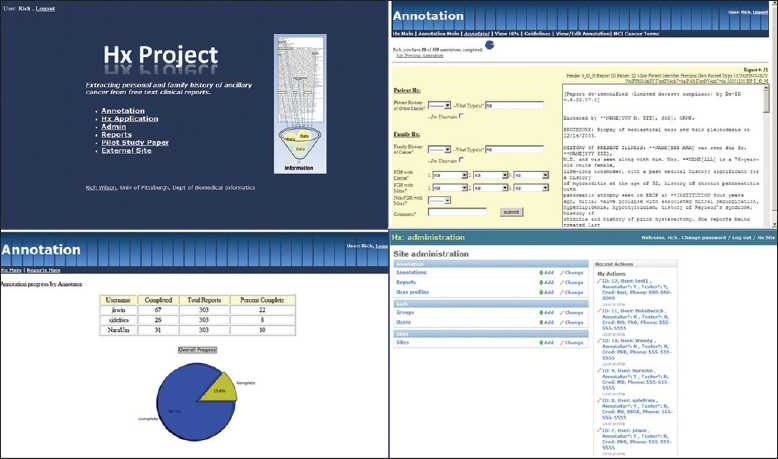
Django web annotation tool. Screen shots of the web annotation tool: main portal, annotation screen, progress report and administrative interface

We created a web-based annotation tool for report annotation and for managing reference standard development. Our tool was built using the Django (www.djangoproject.com) web framework, which is an open-source project built on the Python (www.python.org) programming language. Django provides a scalable platform for rapid web application development using the classic model-viewer-controller design pattern.[15] The annotation tool features include controlled user access, database support, search, progress reporting, task-specific error checking and a site administration interface. The application design focused on providing the user with an intelligent workspace, such as displaying annotation forms and reports with the same view, automatic report queuing and providing easy access to annotation guidelines. Screenshots for the application are provided in Figure 1, or visit http://quiil.dbmi.pitt.edu/hx.

### Hx Application

We developed the Hx application for this project to process HandP reports and classify them according to personal and family history of cancer. Hx was written in Python, which leverages the Natural Language Tool Kit and other libraries to process reports.[16] The application incorporates many NLP techniques, such as semantic frames, hot-spotting on key concepts, a bi-directional dynamic window search, knowledge about report structure and domain-specific lexical knowledge sources. The application reads a report, extracts information needed to make our two classifications (Q1 and Q2), evaluates the information and classifies the report and provides aggregate information for the entire report set. To accomplish our classification tasks, Hx is organized into three primary modules (described below): report parsing, frame building and frame evaluation. There are two key NLP techniques the application leverages heavily that require elaboration before we begin a detailed description of each module.

Semantic frames were used to store information about each cancer concept located in the text. Each cancer frame consisted of seven slots or attributes: report number, the cancer term located (e.g., “mesothelioma,” “cancer,” “tumor”), term modifier (e.g., “malignant,” “lymphocytic,” “lung,” etc.), the experiencer (patient, father, etc.), negation terms, section location and the raw sentence text. During Hx development, it was clear that this classification task hinged on building accurate frames. Simply identifying the word “tumor” in the text tells you little about who is experiencing the cancer, whether or not it is negated and whether or not it is the known mesothelioma or some ancillary cancer.

The second critical technique was the use of a domain-specific lexicon that was used in executing all three modules. The lexicon consists of five categories and was created by the authors during development. First, terms used to assist in cancer concept identification, which include non-cancers (e.g., hematoma), cancer acronyms (e.g., SMLC) and unique cancers (e.g., leukemia). Second, modifying terms consist of descriptors (adjectives) for the cancer concept (e.g., cervical cancer, malignant tumor) and terms, such as “cancer” or “tumor,” which serves as the adjective (e.g., tumor markers, cancer conference). Third, kinship terms include first-degree relatives (e.g., mother, sister), other relatives (e.g., uncle) and non-kinship terms (e.g., spouse). Fourth, report section heading terms include family history section variants (e.g., “family history:”, “FH:”) and non-subjective section headings (e.g., “physical exam,” “assessment and plan”). Finally, negation terms and phrases such as “no history of” and “unremarkable for.” The complete lexicon can be found in Table 1.

**Table 1:** Domain-specific lexicon

Type		Terms
Cancer identification	Noncancers	“adenoma,” “hematoma,” “adenomas,” “cystadenoma,” “hamartoma,” “hematomas,” “glaucoma,” “hemangioma,” “lipoma,” “hemartoma,” “coma,” “diploma,” “aroma”
	Cancer acronyms	“ALL,” “ALCL,” “AMKL,” “ANLL,” “CTCL,” “CLL,” “AML,” “CML,” “HCC,” “HCL,” “LMM,” “TCC,” “T-PLL,” “PLL,” “SCC,” “SMLC,” “SCLC”
	Unique cancers	“hodgkin,” “leukemia,” “neoplasm,” “tumor”
Modifiers	Descriptors	“acute,” “acute lymphoblastic,” “acute myelogenous,” “adrenocortical,” “aids-related,” “anal,” “basal cell,” “bile duct,” “bladder,” “bone,” “brain,” “brain stem,” “breast,” “bronchial,” “central nervous system,” “cerebellar,” “cervical,” “chronic lymphocytic,” “chronic myelogenous,” “colon,” “colorectal,” “cutaneous t-cell,” “endocrine,” “endometrial,” “esophageal,” “eye,” “gallbladder,” “gastric,” “gastrointestinal,” “gastrointestinal carcinoid,” “germ cell,” “hairy cell,” “head,” “hepatocellular,” “hodgkin,” “hodgkin's,” “hypopharyngeal,” “hypothalamic,” “intraocular,” “kaposi,” “kidney,” “laryngeal,” “lip,” “liver,” “lung,” “lymphoblastic,” “lymphocytic,” “malignant,” “melanoma”, “metastatic,” “merkel cell,” “mouth,” “myelogenous,” “myeloid,” “nasal cavity,” “nasopharyngeal,” “neck,” “non-hodgkin,” “non-small cell,” “oral,” “ovarian,” “pancreas,” “pancreatic,” “paranasal,” “parathyroid,” “penile,” “pharyngeal,” “pituitary,” “plasma cell,” “pleuropulmonary,” “prostate,” “rectal,” “renal,” “renal cell,” “salivary gland,” “sinus,” “skin,” “small cell,” “small intestine” “soft tissue,” “spinal cord,” “squamous cell,” “stomach,” “stromal,” “t-cell,” “t-cell prolymphocytic,” “testicular,” “throat,” “thymic,” “thymus,” “thymoma,” “thyroid,” “transitional cell,” “unknown,” “unknown primary site,” “uterine,” “vaginal,” “vulvar,” “wilms”
	Nouns with cancer serving as an adjective	“conference,” “marker,” “markers,” “registry”
Kinship terms	First-degree relatives (FDR)	“mother,” “father,” “sister,” “sisters,” “brother,” “brothers,” “half brother,” “half sister,” “son,” “sons,” “daughter,” “daughters,” “half-brother,” “half-sister,” “step brother,” “step sister,” “step-brother,” “step-sister,” “mom,” “dad,” (step- and half-siblings intentionally included)
	Non-FDRs	“uncle,” “aunt,” “cousin,” “grandfather,” “grandmother,” “grandpa,” “grandma”
	Nonrelatives	“husband,” “wife,” “spouse”
Section headings	Family history sections	“family history,” “family history:”, “familyhistory,” “family history,” “family history\r,” “FH:”
	Nonsubjective headings	“Physical Exam,” “Physical Exam:”, “Physicalexam,” “Physicalexam:”, “PHYSICAL EXAMINATION,” “PHYSICAL EXAMINATION:”, “PHYSICALEXAMINATION,” “PHYSICALEXAMINATION:”, “Assessment,” “ASSESSMENT,” “ASSESSMENT:”, “ASSESSMENT/PLAN,” “ASSESSMENT/PLAN,” “ASSESSMENT/PLAN:”, “ASSESSMENT AND PLAN,” “ASSESSMENT AND PLAN:”, “ASSESSMENT PLAN,” “ASSESSMENT-PLAN,” “ASSESSMENT-PLAN:”, “IMPRESSION,” “IMPRESSION:”
Negation phrases	Negation	“negative,” “negative for,” “denies,” “denies any history of,” “denies any family history of,” “negative history of,” “no family history of,” “no,” “unremarkable,” “unremarkable for,” “no history of,” “no history of other,” “no hx of,” “no family history of,” “no family hx of,” “without a history of,” “without of hx of,” “without”
		
		
		
		

### Report Parsing Module

Hx begins by reading in the source text file, which contained 300 test HandP reports, parsing it into individual reports using text preprocessing, document structure segmentation, regular expression matching and lexicon look-ups. Leveraging Python’s object-oriented inheritance, each report is then processed in turn by creating HandP report, section and sentence objects.

Medical records are generally arranged using the subjective-objective-assessment-plan format developed by Dr. Lawrence Weed in the 1960s.[17] The “subjective” portion typically contains patient-generated information, such as their history. We leverage this document format to limit our search space to focus only on the subjective sections of the report. Using a lexicon of common titles for subjective sections (e.g., “History of Present Illness”), Hx then processes the sentences, within those sections, with the frame building module.

### Frame Building Module

The frame building module identifies cancer concepts in the text and constructs a “cancer frame” consisting of seven slot values. The data placed in these slots are vital to an accurate report classification. Because of the criticality of this task, we explored two methods for determining the frames slot values. Dynamic-Window method is the original approach designed with the Hx application and the second uses ConText, an existing algorithm, to build the frames. Each method of building frames was tested in this study, using the same modules for report parsing and frame evaluation.

### Frame Building Module: Dynamic-Window Method

**Figure 2:**
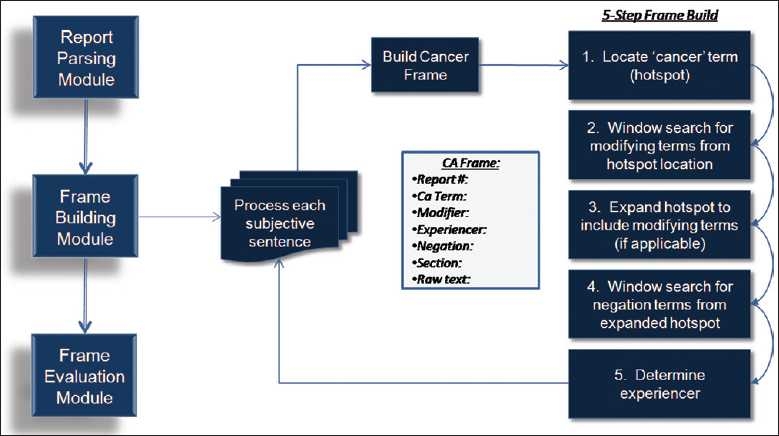
Frame build module: Dynamic-Window method

**Figure 3:**
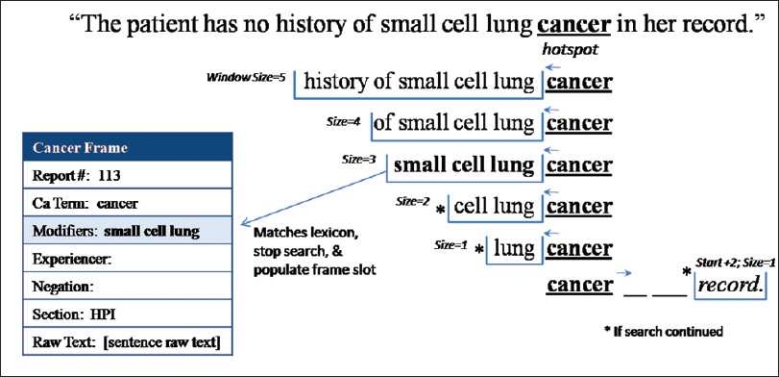
Frame build (step 2): Variable-size bi-directional window search (modifiers). Frame building step 2: variable-size window search for modifying terms from the initial “hot-spot”

The Dynamic-Window frame building method builds cancer frames using a five-step process [Figure 2]. Step 1: the application identifies cancer concepts, called “hot-spots,” in the text using a rules-based approach and the supporting lexicon. Cancer concept identification begins with tokenizing the words in a sentence into individual elements and identifying words with an “-oma” suffix, such as “carcinoma” or “blastoma.” If no “-oma” term is found, then Dynamic-Window searches for the word “cancer” in the text. Then, the application searches for unique cancers (e.g., neoplasm, leukemia) and for cancer acronyms found in the supporting lexicon. For example, in the sentence “*The patient has no history of small cell lung cancer in her record,*” the term “cancer” would be identified [Figure 3] as a cancer concept and marked as our “hot-spot” for future slot value search.

Step 2 identifies terms modifying the cancer using a variably sized, bi-directional window search and adds them to the frame slot – modifiers. Each window consists of size *n* terms that search before and after the “hot-spot.” In our example sentence shown in Figure 3, the window size of 5 returns the five terms before the term “cancer” – “history of small cell lung.” Using the “hot-spot” as our starting position, we first look, in order, at the preceding terms using window sizes of 5, 4, 3, 2 and 1. After the contents of each window are returned, Dynamic-Window evaluates whether or not the entire window matches the modifying terms in the lexicon. If the window does not match, the next window size is evaluated. If no preceding windows return a match, the Dynamic-Window method looks forward three places with a window size of 1 to capture the common pattern “cancer of the *location*.” Figure 3 provides a demonstration of this window search and the words returned by each. Note that in this example, window size 3 returns “small cell lung,” which matches the lexicon [Table 1] thus terminating the modifier search and adding the phrase to the frame slot.

**Figure 4:**
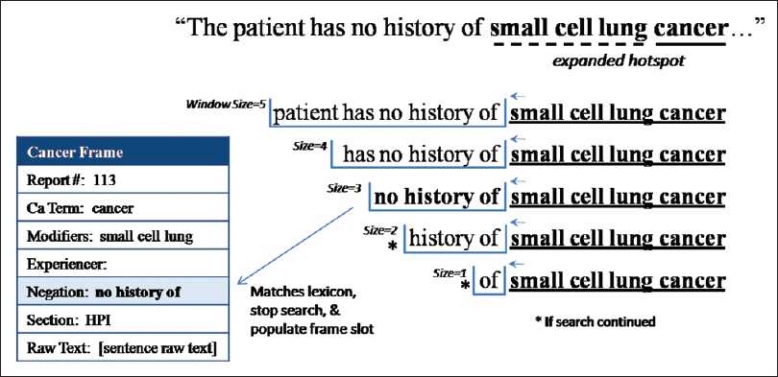
Frame build (step 3): Dynamic-Window search (negation). Frame building step 3: dynamically expand the “hot-spot” and window search for negation

In step 3, if a modifier has been found, Dynamic-Window dynamically expands the “hot-spot” to include the cancer phrase plus any modifiers. In the example sentence [Figure 3], even the largest window size of 5 was not large enough to reach the negation phrase, “no history of,” due to the presence of three modifying terms before the hot-spot location (“cancer”). Without an adjustment, this sentence would lead to an incorrect classification of having a cancer history when none exists. Dynamic-Window accounts for this by expanding the “hot-spot” to include any modifying terms found in step 2 into one unified concept (e.g., small cell lung cancer). Step 4 then uses a window search similar to step 2 (excluding a forward look) to match terms returned by each window size (5, 4, 3, 2 and 1) to the negation phrases found in the lexicon. Figure 4 demonstrates how our “hot-spot” expanded from “cancer” to “small cell lung cancer” and the resulting window search. In this example, the window size of 3 would return the phrase “no history of” and match it to the lexicon. The negation phrase would then be added to the frame. If no modifying terms were found or a modifying term was found forward of the original “hot-spot,” the “hot-spot” would not be dynamically expanded.

The final step completes the frame by determining who is experiencing the identified cancer (e.g., patient or family). Dynamic-Window sets the experiencer to be the patient by default and then tests for other experiencers in the sentence, which, if found, will override the default. To check for other experiencers, Dynamic-Window first leverages the report structure. If the sentence is found in a “family history” section, the experiencer is set to “unknown family.” Next, the Dynamic-Window frame building method checks for the presence of the phrase “family history (of)” in the sentence being processed and then sets the experiencer to “unknown family.” If either of the first two rules is satisfied, Dynamic-Window performs a detailed look into the sentence to locate specific kinship terms (first degree and other relatives) to provide a specific experiencer other than “unknown family.” Finally, if the first two rules (section heading and “family history” phrase) fail, the sentence is evaluated for the presence of specific kinship terms (e.g., son, mother). The frame is disregarded if a nonrelative (e.g. spouse) is located because it is neither the patient nor a blood relative. If a relative from the lexicon is located, the experiencer is set to that relative (e.g., mother, uncle). The frame is now complete and the next subjective sentence is evaluated.

### Frame Building Module: ConText Method

We tested a second frame building method using an existing algorithm, ConText. ConText is an extension of the NegEx algorithm, which uses regular expressions and lexical cues to detect clinical conditions and related modifiers.[18] The NegEx approach previously performed well in identifying negation in discharge summaries.[19] This negation algorithm was then extended to a more general algorithm called ConText to identify historical and hypothetical findings as well as to recognize experiencers other than the patient. Harkema *et al*. tested ConText on various medical records types with moderate success.[20] ConText has been further augmented to determine certainty and quality of exam and was successfully used to evaluate pulmonary embolism findings in radiology exams.[21]

We modified ConText for our task and configured it to build cancer frames from the subjective portions of the HandP reports. We augmented the existing ConText lexicon to include our domain-specific lexicon of findings (cancer concepts), experiencers (e.g., family members), modifiers (e.g., anatomic locations) and negation phrases. ConText then evaluated all the subjective sentences from each report. Once all the information was extracted and the frames had been generated, they were evaluated in the same way as our Dynamic-Window frame building method.

### Frame Evaluation Module

**Figure 5:**
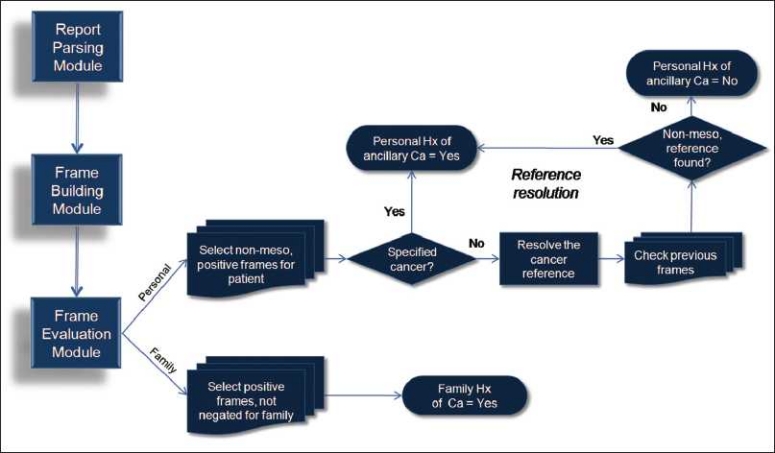
Frame evaluation module and reference resolution

Once all reports have been processed, the frame evaluation module assesses the cancer frames for each report and provides the user with aggregate classification results. Two classifications will be made for each report. First, the patient’s history of ancillary cancer (other than mesothelioma) is determined and, second, the family history of any cancer. For each report, frames are evaluated in succession. To classify a report with regard to a patient’s history of ancillary cancer, we first select frames assigned to a patient in which “mesothelioma” is not the identified cancer concept and the frame is not negated [Figure 5].

As we evaluate frames assigned to the patient, we must remember the need to distinguish between mesothelioma and other cancers. Some frames are clear and have captured a specific cancer from the text, such as “adenocarcinoma” or “lung cancer.” However, natural language is not always that clear. Many frames simply capture the term “cancer” or “tumor,” like in the sentence – “*The cancer was excised 6 months ago.*” We must now perform reference resolution in order to determine whether the phrase “the cancer” is referring to mesothelioma or some other cancer. If an ambiguous cancer concept such as “the cancer” has been identified, Hx performs reference resolution by searching the previous frames, in reverse order, to find a specified cancer like “adenocarcinoma.” If a specified cancer frame is not found, mesothelioma is assigned. The frame is thereby excluded from further evaluation as only nonmesothelioma frames are evaluated (we know they have mesothelioma). Frames with a specified cancer, or frames in which reference resolution yielded a nonmesothelioma reference, trigger classification of this report as positive for personal history of ancillary cancer.

The second classification, family history of any cancer, is relatively uncomplicated. Frames that are assigned to a family member are evaluated in succession. Cancer frames that include any cancer concept (including mesothelioma) and are not negated result in a report classification of positive for family history of cancer [Figure 5].

The final steps of the evaluation provide summary data to the user. Classifications for each report are collected as each report is processed by Hx. Summary data are provided to the user, listing positive reports for each classification.

### Measurements

This study measured the performance of the application using two methods versus the reference standard, with the latter serving as the correct answers for each report. First, true positives (TP), true negatives (TN), false positives (FP) and false negatives (FN) were determined. For example, if report #22 was classified by Hx as positive for a personal history of ancillary cancer and was in agreement with the reference standard, it would yield a TP. The performance was measured by four metrics:

- Accuracy: (TP + TN)/total reports.
- Precision (P): TP/(TP + FP) – measure of exactness.
- Recall (R): TP/TP + FN – measure of completeness.
- F-measure: 2(P*R)/P + R – combination of P and R.

Precision provides us with a measure of exactness and recall provides a measure of completeness. However, in order to test our hypothesis, we decided to use a single statistic to evaluate the algorithms. The F-measure provides the harmonic mean of exactness and completeness. Therefore, to determine whether or not the Hx application performed as well as a human, we compared the application’s F-measure with the average kappa values from our annotators.

## RESULTS

### Reference Standard Agreement

Reference standard development indicated a fairly balanced set of test reports. Of the 300 reports, 20% had a personal history of ancillary cancer indicated in the report and 27% noted a family history of any cancer. These sets will serve as the correct answer for evaluating the algorithm classification.

For this study, we had an excellent agreement between individual annotators for each of the two primary questions [Table 2]. The average pairwise observed agreement was 96.2% and 98.9% for question #1 and #2, respectively. Average positive specific agreement was 90.6% and 97.6%. Average negative specific agreement was 97.9% and 99.2%. As expected, positive specific agreement was lower for both questions due to an unequal prevalence of positive cases. Finally, a kappa statistic was calculated to adjust for chance in our agreement, which was 88.3% and 97.2%. The average Cohen’s weighted kappa will serve as the human equivalent in measuring against our Hx application.

**Table 2:** Annotator agreement. Reference standard agreement among our human annotators

	Annotators	Observed agreement	Specific agreement		Kappa
			Ppos	Pneg	
Q1: Personal history of ancillary cancer?	#1 vs. #2	0.963	0.909	0.977	0.886
	#1 vs. #3	0.963	0.908	0.977	0.885
	#2 vs. #3	0.96	0.902	0.975	0.877
Average		0.962	0.906	0.976	0.883
Q2: Family history of cancer?	#1 vs. #2	0.983	0.969	0.989	0.957
	#1 vs. #3	0.993	0.988	0.995	0.983
	#2 vs. #3	0.99	0.981	0.993	0.975
Average		0.989	0.979	0.992	0.972

### Hx Application Performance

We tested the Hx application’s classification performance on the test set of 300 HandP reports. Each report required two classifications (personal and family), resulting in 600 responses. We tested the application using two separate methods for building semantic cancer frames. Application performance against the reference standard using both methods was excellent. The results of each method on our four performance measures can be found in Table 3. The overall accuracy of Dynamic-Window and ConText methods, on both questions, was identical at 96.2%. All differences between the two methods were quite small. However, Dynamic-Window performed better than ConText with regard to precision and F-measure, but ConText performed better in recall. When we compared our two methods’ F-measures of 91.8% for Dynamic-Window and 91.6% for ConText, we observed that both the methods performed similarly to our human benchmark, the average kappa score of 92.8% from our human annotators.

**Table 3:** Overall algorithm performance with both frame methods. Averaged algorithm performance on both questions versus the reference standard

	Dynamic-Window	ConText
Accuracy	0.962	0.962
Precision	0.893	0.877
Recall	0.944	0.961
F-measure	0.918	0.916
	
Avg kappa (ref std)	0.928	

When we break down performance measures to look at each specific question, we see that Dynamic-Window is able to slightly outperform our human standard. Table 4 illustrates specific performance by each question: Q1 (personal) and Q2 (family). For Q1, Dynamic-Window outperformed ConText in accuracy, precision and F-measure, and ConText again outperformed Dynamic-Window in recall. For Q2, ConText outperformed Dynamic-Window in accuracy, precision, F-measure, and equalled Dynamic-Window in recall. Comparing our question-specific (Q1 and Q2) F-measures to the average kappa, we see that Dynamic-Window beat the human benchmark for Q1, as did ConText for Q2. Confidence intervals for accuracy, precision and recall also indicate similarity in the performance of both methods to each other and versus the human benchmark.

**Table 4:** Specific frame method performance. Frame building performance on each question versus the reference standard

	Q1: Personal history of ancillary cancer?		Q2: Family history of cancer?	
	Dynamic-window	ConText	Dynamic-window	ConText
Accuracy	**0.957**	0.937	0.967	**0.987**
	(0.927, 0.975)	(0.903, 0.959)	(0.940, 0.982)	(0.966, 0.995)
Precision	**0.885**	0.789	0.900	**0.964**
	(0.782, 0.943)	(0.680, 0.868)	(0.821, 0.947)	(0.900, 0.988)
Recall	0.900	**0.933**	**0.988**	**0.988**
	(0.799, 0.953)	(0.841, 0.974)	(0.934, 0.998)	(0.934, 0.998)
F-measure	**0.893**	0.855	0.942	**0.976**
Avg kappa (ref std)	0.883		0.972	

## DISCUSSION

Our study tested the ability of an automated system to extract mesothelioma patients’ personal history of ancillary cancer and family history of any cancer from free-text clinical reports. To test this hypothesis, Hx identifies cancer concepts in the text and then builds semantic frames from extracted values from the surrounding text, such as cancer modifiers, the experiencer and any negation terms. In doing so, we tested two specific methods for constructing our semantic frames. One, the Dynamic-Window method, is a rule-based algorithm that constructs semantic cancer frames through hot-spotting on key concepts, a bi-directional dynamic window search and a domain-specific lexical knowledge source. The second method used modified the existing ConText algorithm to build cancer frames. Each method of building frames was tested in this study using the same modules for report parsing and frame evaluation. The frame evaluation module provides critical reference resolution for unspecified cancer concepts (e.g., “the cancer”) and delineation between the known cancer (mesothelioma) and ancillary cancer of interest.

We tested Hx using both frame building methods against a human-annotated reference standard developed from a test set of 300 HandPs from mesothelioma patients. To test our hypothesis, we compared the reference standard’s average Cohen’s weighted kappa against each algorithm’s performance (F-measure). Hx proved to have similar performance to our human benchmark. Both of our two methods resulted in a surprisingly high accuracy, both scoring 96.2%, and outperformed the human benchmark when F-measures were compared with average Kappas. For the personal history question, Dynamic-Window scored an 89.2% against the human benchmark of 88.3% and for the family history question, ConText outperformed the benchmark, with an F-measure of 97.6% versus 97.2%.

### Reference Standard

Agreement among annotators is an indication of the quality of a reference standard. Agreement for the test set was very high, particularly for the family history question. The average kappas from the reference standard were much higher than in our initial pilot study of 100 reports. The increase in annotator agreement is intriguing and suggests the influence of one or more changes from the pilot study. First, two of our three annotators were changed from the pilot to the test study due to scheduling and experience. Second, the use of a web-based annotation tool provided some assistance to annotators. In the pilot study, annotators were forced to toggle between an MS Excel spreadsheet, text files of reports and a separate file of documentation guidelines to perform annotations. For the test set, our Django web-based application was built for this annotation work. This web application provided a user-centered work space, with the annotation form and report all in one screen. It also provided easy access to previous annotations, documentation guidelines and examples without requiring closing their current workspace (e.g., window). The application’s error checking rules limited user errors. Third, the researcher’s experience from the pilot study reference standard development generated some valuable lessons learned in developing the reference standard for the test set.

### Error Analysis

Hx performed two classifications on 300 clinical reports using two separate methods. As the modules for report parsing and frame evaluation were identical, much of our discussion here will focus on the methods for extracting information for our frames. As indicated by the identical accuracy score (96.2%), each method had a total of 23 errors out of 600 responses each. Table 5 shows the number of errors for each method by question and by type of error (e.g., FN, FP). The most frequent error types were ConText’s 15 FPs on Q1 and Dynamic-Window’s nine FPs on Q2.

**Table 5:** Error analysis. Errors for each question by algorithm and error type

		Dynamic-window		ConText	
Q1: Personal (out of 300)	False positves	7	(0.023)	15	(0.050)
	False negatives	6	(0.020)	4	(0.013)
Q2: Family (out of 300)	False positves	9	(0.030)	3	(0.010)
	False negatives	1	(0.003)	1	(0.003)
	Total (out of 600)	23	(0.038)	23	(0.038)

A total of 46 errors were generated by both methods. Analysis revealed 34 of these errors to be unique, as 12 reports were errantly classified by both Dynamic-Window and ConText. For example, both methods falsely classified report 62 as “no personal history” (Q1), creating an FN for each. There were 11 errors unique to Dynamic-Window and 11 for ConText. Table 6 presents the number of unique errors per algorithm by type along with errors shared by both. No report was incorrectly classified for both Q1 and Q2 by either method, which indicates that a voting scheme with the two methods could be beneficial.

**Table 6:** Unique errors by frame method. Number of unique errors for each frame building method and errors common to both

	Unique to	Dynamic-window	Con Text	Error in both
Q1: Personal (out of 300)	False positves	1	9	6
	False negatives	2	0	4
Q2: Family (out of 300)	False positves	8	2	1
	False negatives	0	0	1
	Total (out of 600)	11	11	12

Careful analysis of each specific error illuminated potential improvements that could be made to the application. Both frame building methods appear to suffer from deficiencies in the supporting lexicon, resulting in 17 out of the 46 total errors. The lexicon deficiency had a greater effect on ConText, which generated 11 of the 17 lexicon errors. Overall, these errors were primarily due to terms or phrases that were not found in the training set but appeared in the test set. For example, in our test set of reports, the phrase “tumor debulking” is found several times. From this phrase, a modifier “debulking” is not a term of importance for either method and thus a “tumor” frame is built when it should be ignored. The positive side of a lexicon deficiency is that it can easily be remedied and continually be improved.

Simple changes to the lexicon would correct 17 errors between the two frame building methods. The result would be an overall F-measure improvement for the Dynamic-Window method from 91.8% to 93.8% and from 91.6% to 95.7% for ConText. Table 7 displays the before and after effect of the lexicon improvements on our four performance measures. All measures improved for each method and the new F-measures demonstrate both methods’ ability to outperform our human benchmark of 92.8%.

**Table 7:** Performance with improved lexicon. Posttest performance for each frame building method with lexicon improvement

	Dynamic-window		ConText	
	Original test	With improved lexicon	Original test	With improved lexicon
Accuracy	0.962	0.972	0.962	0.980
Precision	0.893	0.898	0.877	0.919
Recall	0.944	0.982	0.961	1.000
F-measure	0.918	0.938	0.916	0.957
Avg kappa (ref std)	0.928			

The Dynamic-Window method had 17 other errors that were not categorized as lexicon deficiencies. Seven of these remaining errors were the result of the search window size, particularly in its effort to correctly tackle negation in a sentence. While the starting position of the window is dynamically offset based on modifying terms, the window size itself cycles over fixed values. For example, in report 113, the sentence “there is no family history of diabetes or cancer.” The inclusion of the preposition “of diabetes” in this sentence places the negation phrase out of reach from Dynamic-Window’s largest window size of five words. However, the fixed window sizes do prevent the Dynamic-Window method from reaching too far from the hot-spot and grabbing unrelated terms. It is not known whether a fix (window size increase) for this error would generate additional errors. A potential solution would be to apply a syntactic parse to identify the scope of the negation phrase. Additionally, Dynamic-Window has a problem with multiple cancer terms appearing in the same sentence. This problem only accounted for one error, but a new data set would certainly generate more.

ConText was most clearly affected by lexicon deficiencies. While Dynamic-Window may not go back far enough to find negation, ConText suffers from going too far. In six of the lexicon errors unique to ConText, the error occurred not only because “debulking” was not recognized as a modifying term but also because a false modifying term was found. In the sentence “*patient underwent tumor debulking with chemoperfusion, splenectomy, omentectomy and interoperative ultrasound of the liver,*” ConText located “tumor,” ignored “debulking,” but then located “liver” at the end of the sentence. Therefore, it errantly created a “liver tumor” frame. Dynamic-Window gets this correct because its window size never exceeds five in any direction and “liver” was not located. In the future, ConText could be further modified to apply different scoping rules for modifiers. ConText errors are also generated when proper sentence boundaries are not found in the report. For example, two reports had sentences that lacked normal sentence ends in some sections. Therefore, the sentence splitter failed to split at appropriate locations. ConText simply continued searching within the bounds of what it understood to be a sentence and errantly found modifiers such as modifiers or experiencer terms that generated errors.

There were several remaining errors that were common to both applications. Report transcription mistakes or misspellings resulted in both methods falsely classifying two reports. One error, for each method, was the result of an incorrect reference standard answer for a report in which two of the three annotators missed “*TCC*” (transitional cell carcinoma) as a cancer concept. Finally, the “either/or” problem created errors for each method. For example, in the sentence – “*The pathology was consistent with either mesothelioma versus signet-ring cell adenocarcinoma,*” both algorithms were unable to handle the inconclusive nature of this sentence. In this case, frames for both cancer terms were created.

### Limitations

Our study could have benefited from a broader set of HandP reports. Because of the low-incidence rate of mesothelioma, we were limited in the number of unique patients available. The test set included 70 unique patients, with a median of three reports per patient. A more robust would have provided increased exposure to various patient histories and, presumably, include a larger set of physician report authors.

### Applicability and Future Work

NLP could prove to be an increasingly valuable tool in clinical research environments. Automated tools like Hx could be used for case finding or screening existing patient populations. In cancer registry operations, Hx could be applied to automatically retrieve the patient cancer history information in a matter of seconds on an individual report. Abstractors could then spend their time performing quality assurance tasks rather than pouring over multiple HandPs per patient.

Future work would include algorithm improvements to each for identified errors, exploration of the complete annotation set and mapping the lexicon to a specific ontology (e.g., UMLS) of disease concepts. Additionally, the Hx framework could support an expansion of data extraction tools for additional data fields.

## CONCLUSION

We set out to determine whether an automated application could correctly identify a mesothelioma patient’s personal and family history of cancer from clinical reports as well as a human. To do so, the algorithm must distinguish between the known mesothelioma and other ancillary cancers. It must also be capable of identifying all cancer concepts, recognize negation and determine the experiencer (patient, family or other) of an identified concept. The Hx application was developed to extract information and classify clinical reports. We tested two methods (Dynamic-Window and ConText) for performing the central task of building cancer frames, in which both methods were able to outperform our human benchmark. We showed that the more general algorithm, ConText, built cancer frames as well as our task-specific algorithm and, with improvements, would outperform Dynamic-Window. Although Dynamic-Window could be modified to retrieve other concepts, ConText is far more robust and performs better on complex sentences and inconclusive concepts. It was clear that both algorithms were critically dependant on the domain-specific lexicon and negation extraction. Improvements to both would lead to near-perfect results on this data set. These methods could greatly improve and expedite the process of extracting data from free-text, clinical records for a variety of research or healthcare delivery organizations.
